# Facile and high-efficient immobilization of histidine-tagged multimeric protein G on magnetic nanoparticles

**DOI:** 10.1186/1556-276X-9-664

**Published:** 2014-12-10

**Authors:** Jiho Lee, Jeong Ho Chang

**Affiliations:** 1 Korea Institute of Ceramic Engineering and Technology, Seoul 153-801, South Korea

**Keywords:** Magnetic nanoparticle, Immobilization, Protein G, Histidine-tag, Efficiency

## Abstract

This work reports the high-efficient and one-step immobilization of multimeric protein G on magnetic nanoparticles. The histidine-tagged (His-tag) recombinant multimeric protein G was overexpressed in *Escherichia coli* BL21 by the repeated linking of protein G monomers with a flexible linker. High-efficient immobilization on magnetic nanoparticles was demonstrated by two different preparation methods through the amino-silane and chloro-silane functionalization on silica-coated magnetic nanoparticles. Three kinds of multimeric protein G such as His-tag monomer, dimer, and trimer were tested for immobilization efficiency. For these tests, bicinchoninic acid (BCA) assay was employed to determine the amount of immobilized His-tag multimeric protein G. The result showed that the immobilization efficiency of the His-tag multimeric protein G of the monomer, dimer, and trimer was increased with the use of chloro-silane-functionalized magnetic nanoparticles in the range of 98% to 99%, rather than the use of amino-silane-functionalized magnetic nanoparticles in the range of 55% to 77%, respectively.

## Background

Magnetic nanoparticles (MNPs) have been widely used in various biomaterial applications, such as the immobilization of enzymes and proteins, drug delivery systems, labeling and sorting of cells, and the purification of DNA and proteins [1-8]. One of the most recent applications being investigated extensively is the use of functionalized MNPs as magnetic carriers for specific protein purification [9-14]. Among them, immobilization methods for purification specific protein have used metal ion affinity-tagged protein, such as histidine (His)-tagged proteins. The functionalized magnetic nanoparticles and tagged protein offer several advantages for protein purification, such as easy handling and simple procedures. The protein purification can be carried out in a single tube without expensive instruments, centrifuges, or other such systems.

**Figure 1:**
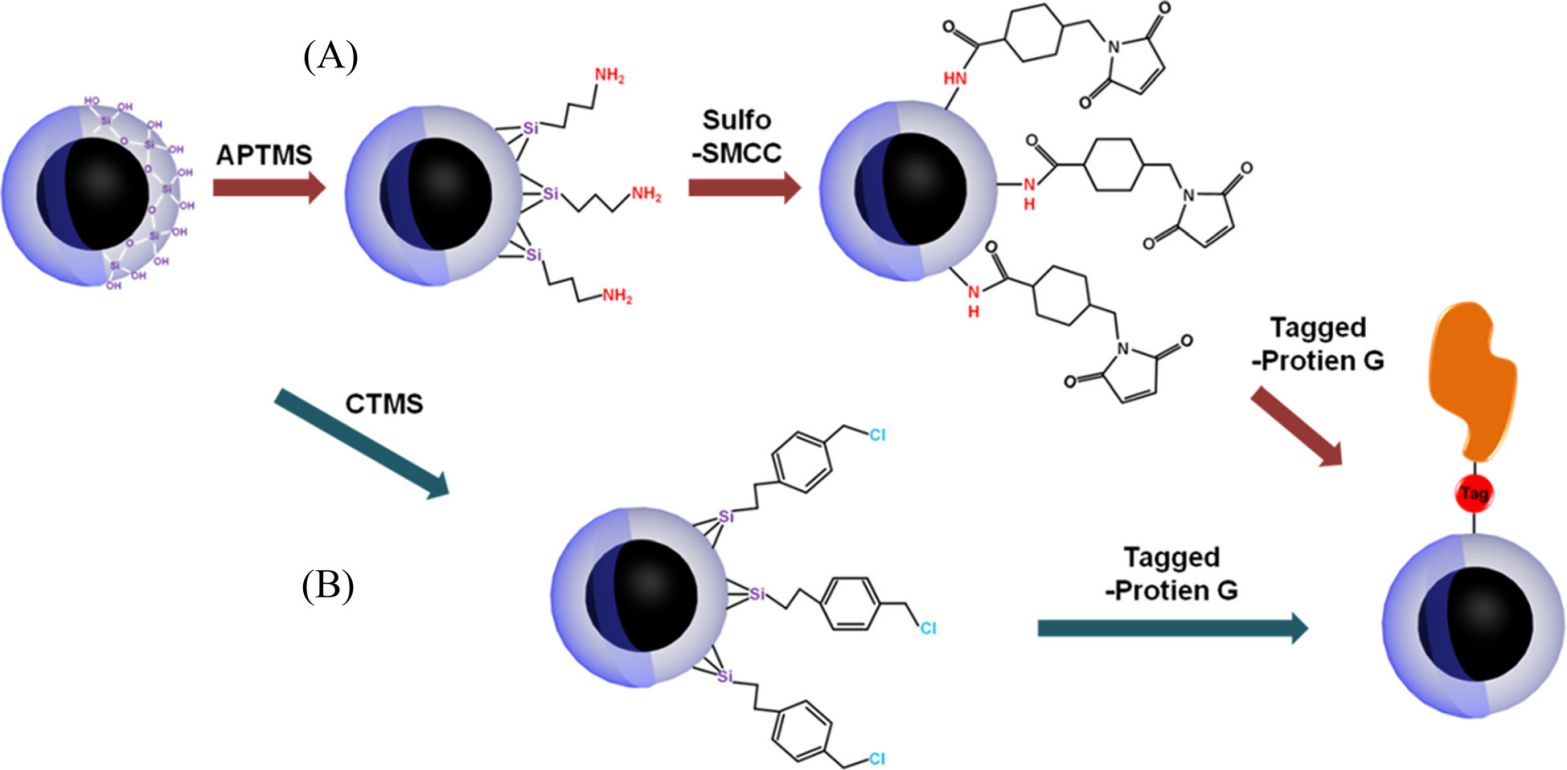
**The schematic diagram of the formation process. (A)** NH_2_-MNPs@SiO_2_ and **(B)** CTMS-MNPs@SiO_2_.

In the past few decades, Xu et al. modified Fe_2_O_3_ magnetic nanoparticles with dopamine compounds and nickel nitrilotriacetic acid (Ni-NTA) to separate His-tagged proteins [15]. Yeh et al. demonstrated that the methods can bind Ni-NTA onto Fe_3_O_4_-NH_3_^+^ nanoparticles for protein purification and cell targeting [16]. Moreover, Li et al. have successfully prepared Fe_3_O_4_ magnetic nanoparticles with immobilized NTA, which were capable of producing Ni(II) ions [17]. However, the methods described above require a difficult and complicated procedure to prepare Ni-NTA-modified magnetic nanoparticles to purify His-tagged proteins. In addition, Lee et al. prepared the functionalized silica-coated magnetic nanoparticles to bind multimeric protein G [18]. The detailed synthesis procedure for functionalized silica-coated magnetic nanoparticles is shown in Figure 1(A). The surface of MNPs was coated with silica to prepare MNPs@SiO_2_. In the first step, amino-functionalized MNPs@SiO_2_ (NH_2_-SiO_2_-MNP) was prepared by incorporating 3-aminopropyltrimethoxysilane (APTMS) on the surface of SiO_2_-MNP. Subsequently, the surface of NH_2_-SiO_2_-MNP was modified with the sulfhydryl group and sulfosuccinimidyl 4-(*N*-maleimidomethyl) cyclohexane-1-carboxylate (Sulfo-SMCC) for coupling with multimeric protein G. The binding efficiency of the monomer, dimer, and trimer protein G were 77%, 65%, and 55%, respectively. However, the procedure used a lot of reagents and was cumbersome, thus increasing the cost. Therefore, we need a novel, simple, and low-cost synthesis method to prepare functionalized MNPs@SiO_2_.

In this work, we report the one-step preparation of multimeric protein G with a His-tagged (His-tag) monomer, dimer, and trimer on magnetic nanoparticles for high-efficient immobilization. This immobilization was demonstrated by two different pathways of amino-silane and chloro-silane functionalization on silica-coated magnetic nanoparticles. To calculate the efficiency, bicinchoninic acid (BCA) assay was used and confirmed by sodium dodecyl sulfate polyacrylamide gel electrophoresis (SDS-PAGE).

## Methods

### Synthesis of the CTMS-MNPs@SiO_2_

Iron (II) chloride tetrahydrate (99%), iron (III) chloride hexahydrate (98%), tetraethyl orthosilicate (98%), and phosphate-buffered saline (PBS) were purchased from Sigma-Aldrich chemical (St. Louis, MO, USA). (Chloromethyl) phenylethyltrimethoxysilane (CTMS) was purchased from Gelest (Morrisville, PA, USA). Pierce BCA™ protein assay kit was purchased from Thermo Scientific Inc. (Pittsburgh, PA, USA). HPLC-grade solvents were used in both the reaction and washing steps. All aqueous solution was prepared with double-distilled water.

MNPs were prepared by coprecipitation with Fe^2+^ and Fe^3+^. To prepare the MNPs, a mixture of 2 M FeCl_2_ · 4H_2_O and 1 M FeCl_3_ · 6H_2_O was added to 0.7 M ammonia solution with vigorous stirring at room temperature. The obtained MNPs were separated by magnet, and the supernatant was decanted. The collected MNPs were washed several times with ethanol and then air-dried. In order to synthesize the silica coating of MNPs, the 100 mg of MNPs were dispersed in 100 mL of cyclohexane under sonication for 3 h in the presence of 3.3 mL of oleic acid. The 44 g of Igepal CO-520 (Sigma-Aldrich chemical) dissolved solution in 900 mL of cyclohexane was added to MNP solution. After stirring for 15 min, 8 mL of aqueous ammonia solution was added dropwise. The mixed solution was further stirred for 20 h at room temperature. Methanol was added to the solution to form dark precipitates, which were collected through magnetic separation after the removal of supernatants. The dark precipitate was washed repeatedly with ethanol and then vacuum dried.

The MNPs@SiO_2_ was then modified with silane coupling agent to introduce chloride group. Briefly, 100 mg of MNPs@SiO_2_ was dispersed in 9 mL of acetone containing 1 mL of CTMS, and the mixture was rotated at room temperature for 12 h in the dark. After silanization, the resultants were separation by magnetic attraction and washed repeatedly with acetone to remove unreacted silane coupling agent, and then dried at room temperature overnight in the dark.

### Immobilization of histidine-tagged multimeric protein G

His-tagged recombinant dimer and trimer of protein G were overexpressed in *Escherichia coli* BL21 by the repeated linking of protein G monomers with flexible (GGGGS)_3_((glycine/glycine/serine)_3_) [18]. The sizes for His-tagged monomer, dimer, and trimer protein G were 16, 32, and 46 kDa, respectively.

The immobilization of His-tagged multimeric protein G experiments were carried out in 200 μL of protein solution dissolved in 10 mM phosphate buffer (pH 7.2) and were added to disperse the chloride group functionalized MNPs@SiO_2_ (5 mg). The initial concentration of protein G was 100 μg/mL. The reaction mixtures were incubated within a rotator at room temperature for 1 h with continual agitation to the suspending particles. After removal of particles by magnetic separation, the multimeric protein G concentrations in the supernatants were determined by fluorescence intensity using a microplate reader (Infinite M200, Tecan Ltd; Männedorf, Switzerland) at 595 nm. The fluorescence intensity of sample was measured five times with a 20-μs integration time by using a microplate reader and BCA protein assay kit.

SDS-PAGE of the samples were performed using standard protocols for conformation of protein binding efficiency [19]. Samples were incubated at 100°C for 5 min in a sample buffer and then separated by SDS-PAGE (12% gels).

### Instrumentation

The particle size and morphology of silica-coated magnetic nanoparticles were determined by transmission electronic microscopy (TEM) using a JEM-4010 (JEOL; Tokyo, Japan) at an accelerating voltage of 400 kV. The magnetic nanoparticles and silica-coated magnetic nanoparticles were characterized by powder X-ray diffraction (XRD), using a Rigaku D/MAX-2500 diffractometer (Rigaku; Tokyo Japan) using filtered Cu Kα radiation, and the data were collected for 2*θ* of 20.0° to 80.0°. The magnetization of silica-coated magnetic nanoparticles at room temperature up to 10 kOe was measured by vibrating sample magnetometer (VSM) using a VSM 4179 (Oxford Instruments; Oxfordshire, UK). Fourier transform infrared (FT-IR) spectroscopy was used to identify the functionalized MNPs@SiO_2_. The FT-IR spectra were recorded on a V-460 (JASCO; Easton, MD, USA) FT-IR spectrometer using KBr pellets. Spectra were obtained at a resolution of 4 cm^-1^, and the wavenumber range from 4,000 to 650 cm^-1^. The fluorescence intensity and absorbance of the samples were measured using microplate reader Infinite M200 (Tecan Ltd). The fluorescence was measured five times for each sample with a 20-μs integration time.

## Results and discussion

Figure 1 shows the synthetic procedures of conventional preparation with (a) a sulfo-SMCC cross-linker from amino-silaneMNPs@SiO_2_ and (b) a one-step preparation with a chloro-silane functional group on MNPs@SiO_2_. This CTMS strongly anchors the MNPs@SiO_2_ surface, while the chloro functions on the outer surface induce coupling between the MNPs@SiO_2_ and His-tagged biomolecules. This method was simple and low cost compared to the Sulfo-SMCC-MNPs@SiO_2_.

**Figure 2:**
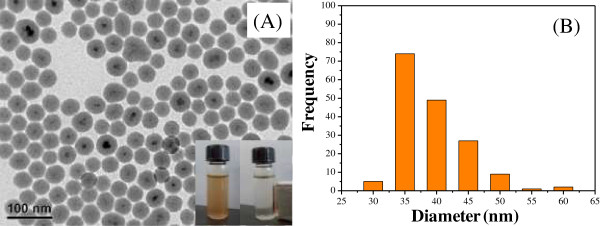
**Typical TEM images and particle size distributions of the MNPs@SiO**_**2**_**. (A)** TEM image and the **(B)** particles size histogram of MNPs@SiO_2_.

The typical TEM images and particle size distributions of the MNPs@SiO_2_ are shown in Figure 2, in which the core/shell structures have a narrow size distribution of 33 to 44 nm. In addition, the mean diameter of the silica-coated MNPs measured from TEM was about 38.9 nm with a narrow size distribution (Figure 2B). The MNPs@SiO_2_ were dispersed in water by ultrasonication, and then easily separated with a magnet within 1 min (inset of Figure 2A).

**Figure 3:**
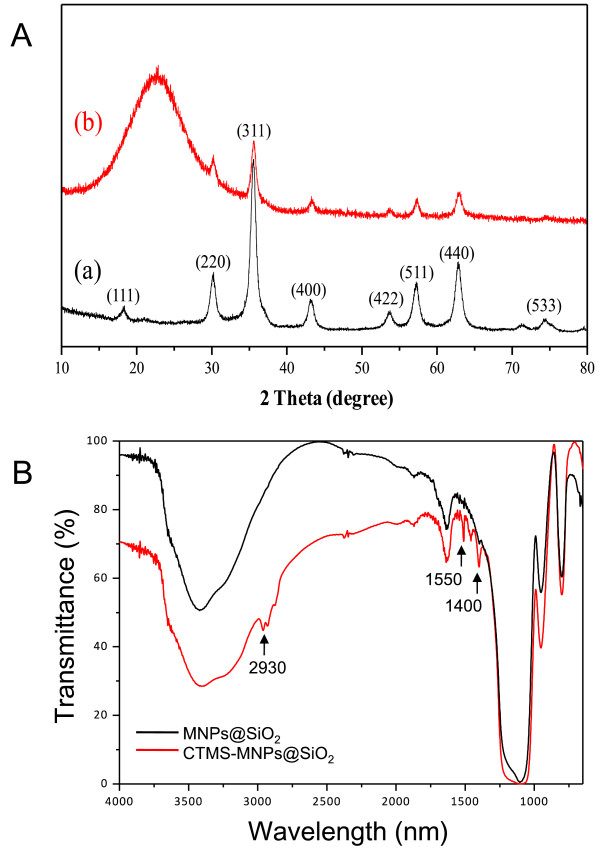
**Wide angle XRD patterns and FT-IR spectroscopy. (A)** XRD patterns of (a) MNPs and (b) MNPs@SiO_2_ and **(B)** FT-IR spectra of MNPs@SiO_2_ and CTMS-MNPs@SiO_2_, respectively.

Figure 3A shows the wide angle XRD patterns of MNPs and MNPs@SiO_2_, in which the characteristic peaks of Fe_3_O_4_ nanoparticles are seen at 30.13°, 35.57°, 43.21°, 53.68°, 57.27°, 62.83°, and 74.29° of 2*θ* corresponding to (220), (311), (400), (422), (511), (440), and (533), respectively. All the diffraction peaks could be indexed to well-crystallized Fe_3_O_4_ (JCPDS no. 19-629) [20,21]. The MNPs were mainly composed of an inverse cubic spinel structure of magnetite. Figure 3(B) shows the broad peak at a low diffraction angle of 20° to 30°, which corresponds to the amorphous state SiO_2_ shells surrounding the MNPs. We were able to find that powder XRD patterns show a very low intensity for the peaks attributed to MNP cores. This was due to the coating of the amorphous silica shell.

In order to confirm the modification of the MNPs' surface, the FT-IR spectroscopy of the prepared MNPs@SiO_2_ and CTMS-MNPs@SiO_2_ materials was obtained (Figure 3B). The bands at 3,400 to 3,500 cm^-1^ were due to -OH stretching on silanol. The peaks at 2,930 cm^-1^ were attributed to the stretching vibration of C-H in aromatic and C-H in alkyl [22]. The peaks at 1,400 to 1,550 cm^-1^ were assigned to the stretching vibration of C-C in an aromatic ring. The presence of additional absorption bands at 800, 950, and 1,110 cm^-1^ were most likely due to the symmetric and asymmetric stretching vibration of framework and terminal Si-O groups. These all confirm that CTMS has been successfully conjugated on the surface of the MNPs@SiO_2_.

**Figure 4:**
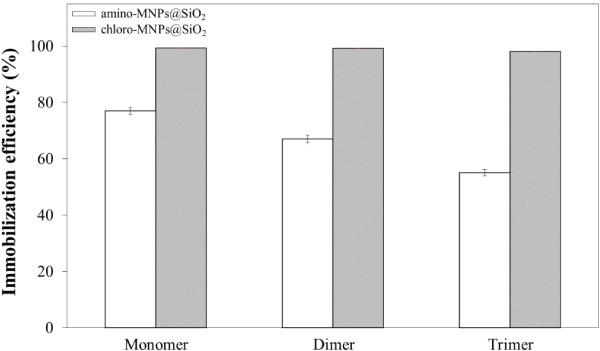
**Immobilization efficiency of His-tagged multimeric protein G on the NH**_**2**_**-MNPs@SiO**_**2 **_**and CTMS-MNPs@SiO**_**2**_**.**

**Figure 5:**
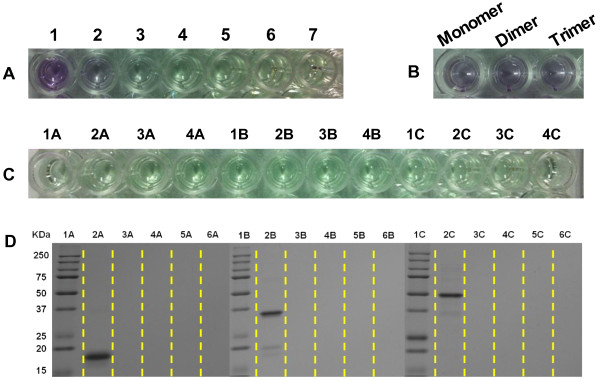
**Photo of concentrations and SDS-PAGE analysis of multimeric proteins.** The photo of **(A)** the BSA standards at the following concentrations (μg/mL) for 1–7, respectively, 200, 100, 50, 25, 12.5, 6.25, and 0; **(B)** the multimeric protein G (100 μg/mL); **(C)** supernatants after binding reaction for the CTMS-MNPs@SiO_2_ with His-tagged monomer, dimer, and trimer protein G, respectively, 1A, 1B, and 1C, and the function washed off from the His-tagged multimeric protein G on the CTMS-MNPs@SiO_2_, respectively, 2A-4A, 2B-4B, and 2C-4C; and **(D)** SDS-PAGE analysis of multimeric protein: lanes M marker; lanes 1A, 1B, and 1C: His-tagged monomer, dimer, and trimer protein G, respectively; lanes 2A, 2B, and 2C: supernatants after binding reaction for the CTMS-MNPs@SiO_2_ with His-tagged monomer, dimer, and trimer protein G, respectively; lanes 3A-5A, 3B-5B, and 3C-5C: the function washed off from the His-tagged multimeric protein G on the CTMS-MNPs@SiO_2_.

Smith et al. developed a bicinchoninic acid assay that was part of colorimetric protein assay techniques [23,24]. The BCA protein assay was used to determine protein concentrations. This assay was based on a Biuret reaction where the presence of protein in an alkaline medium reduces Cu^2+^ to Cu^1+^. The reaction product was formed by the (BCA)_2_ and Cu^1+^ and exhibited a strong absorbance at 562 nm. The protein concentration was reflected by the products changing color from green to purple, which depended on the given protein concentration. To investigate the His-tagged multimeric protein G immobilization efficiency of chloro-silane and amino-silane-functionalized MNPs@SiO_2_, we used the monomer, dimer, and trimer of protein G as a model protein. Figure 4 shows the His-tagged multimeric protein G immobilized quantities on NH_2_-MNPs@SiO_2_ and CTMS-MNPs@SiO_2_, which were determined by a BCA protein assay kit and microplate reader (Figure 5A,B,C). The immobilization efficiency of the His-tagged monomer, dimer, and trimer protein G were observed to be 77.2 ± 1.24%, 67 ± 1.37%, and 55 ± 1.13% for NH_2_-MNPs@SiO_2_, and 99 ± 0.015%, 99 ± 0.006%, and 98 ± 0.005% for CTMS-MNPs@SiO_2_, respectively. The immobilization efficiency of CTMS-MNPs@SiO_2_ material was drastically enhanced rather than that of the NH_2_-MNPs@SiO_2_ because the chloro functions on the outer surface induced coupling between the MNPs@SiO_2_ and His-tagged biomolecules. Furthermore, the specificity of CTMS-MNPs@SiO_2_ nanoparticles binding with His-tagged multimeric protein G was analyzed by SDS-PAGE (Figure 5D). The amount of His-tagged multimeric protein G released from CTMS-MNPs@SiO_2_ nanoparticles was almost negligible (Figure 5D, lines 4A-6A, 4B-6B, and 4C-6C), indicating that the His-tagged multimeric protein G did not bond to the MNPs@SiO_2_ nanoparticles by physical adsorption. It was further demonstrated that His-tagged multimeric protein G could be adsorbed on the surface of CTMS-MNPs@SiO_2_ nanoparticles through an interaction between the His-tags and chloride groups, rather than the physical adsorption effects.

## Conclusions

In summary, a facile and high-efficient one-step immobilization of His-tagged multimeric protein G on the magnetic nanoparticle surface was achieved with two kinds of APTMS and CTMS silanes. Based on the BCA test, the immobilization efficiencies of the His-tagged multimeric protein G with CTMS-MNPs@SiO_2_ were higher than those of NH_2_-MNPs@SiO_2_. The immobilization efficiency with CTMS-MNPs@SiO_2_ nanoparticles was 99.4 ± 0.015%, 99.2 ± 0.006%, and 98.1 ± 0.005% for the His-tagged monomer, dimer, and trimer of protein G, respectively.

## Competing interests

The authors declare that they have no competing interests.

## Authors’ contributions

JL carried out the experiments and participated in the drafting of the manuscript. JHC conceived and designed the study. Both authors read and approved the final manuscript.
